# Transarterial embolization/chemoembolization therapy for hepatocellular carcinoma fed by adrenal artery: Preliminary results: Erratum

**DOI:** 10.1097/MD.0000000000007451

**Published:** 2017-06-30

In the article, “Transarterial embolization/chemoembolization therapy for hepatocellular carcinoma fed by adrenal artery”,^[1]^ which appeared in Volume 95, Issue 52 of *Medicine*, the editor should have been listed as Shizhang Ling instead of Edoardo Villani.
